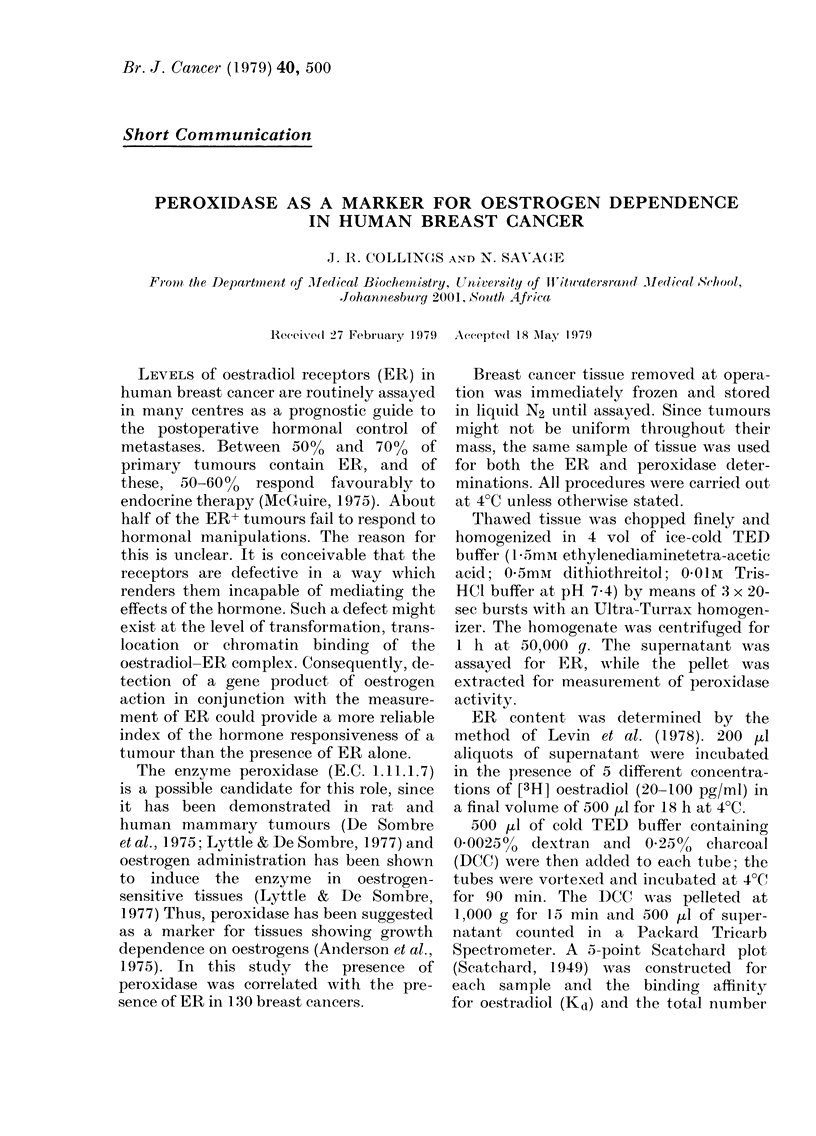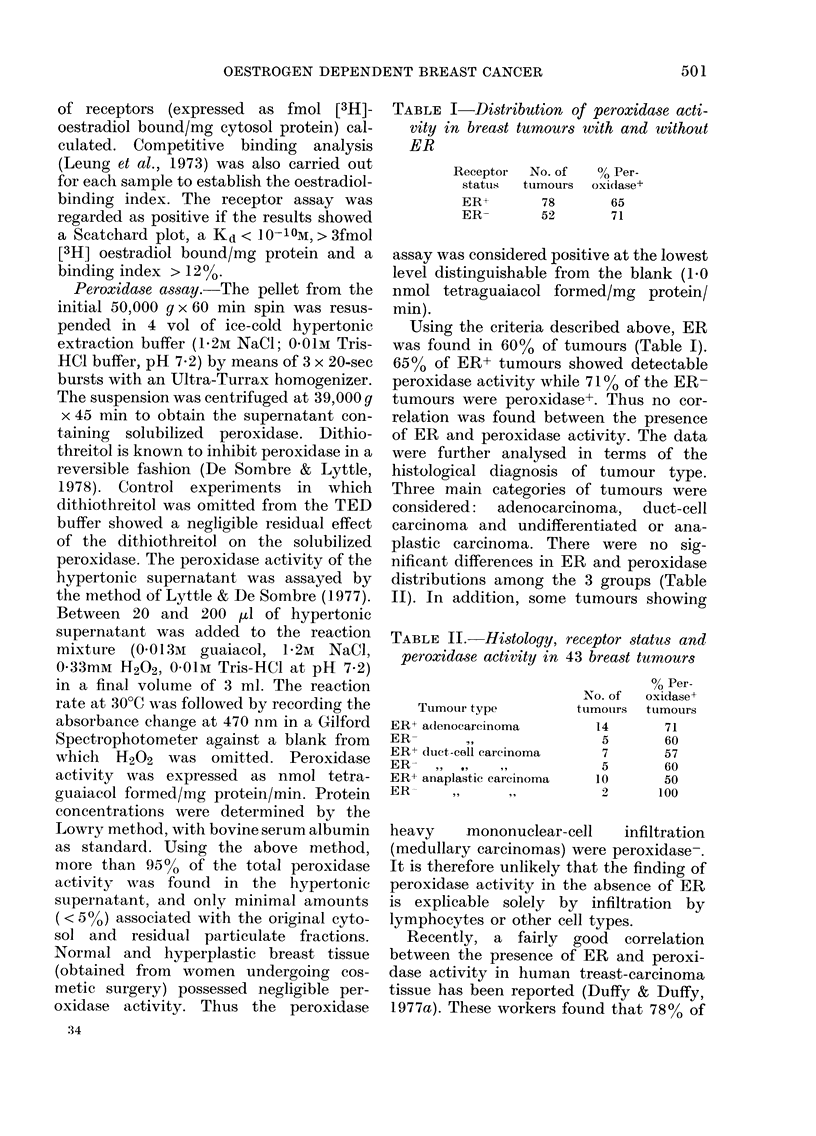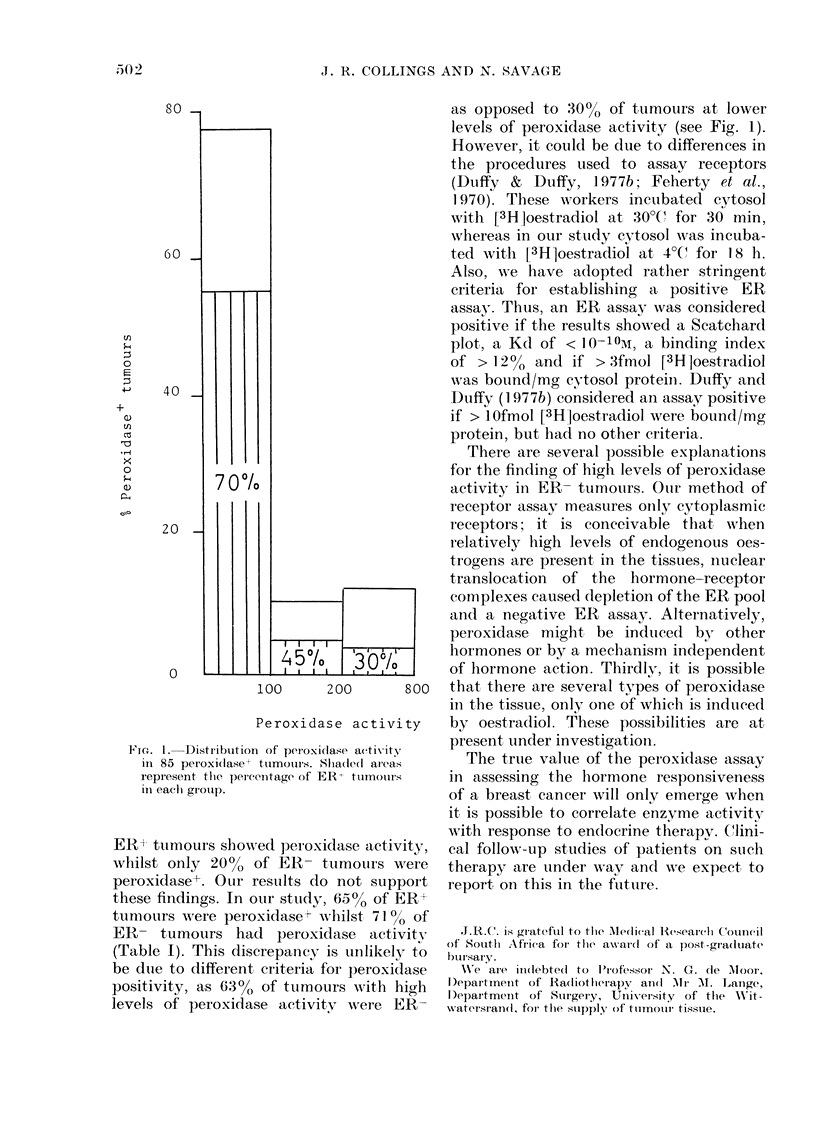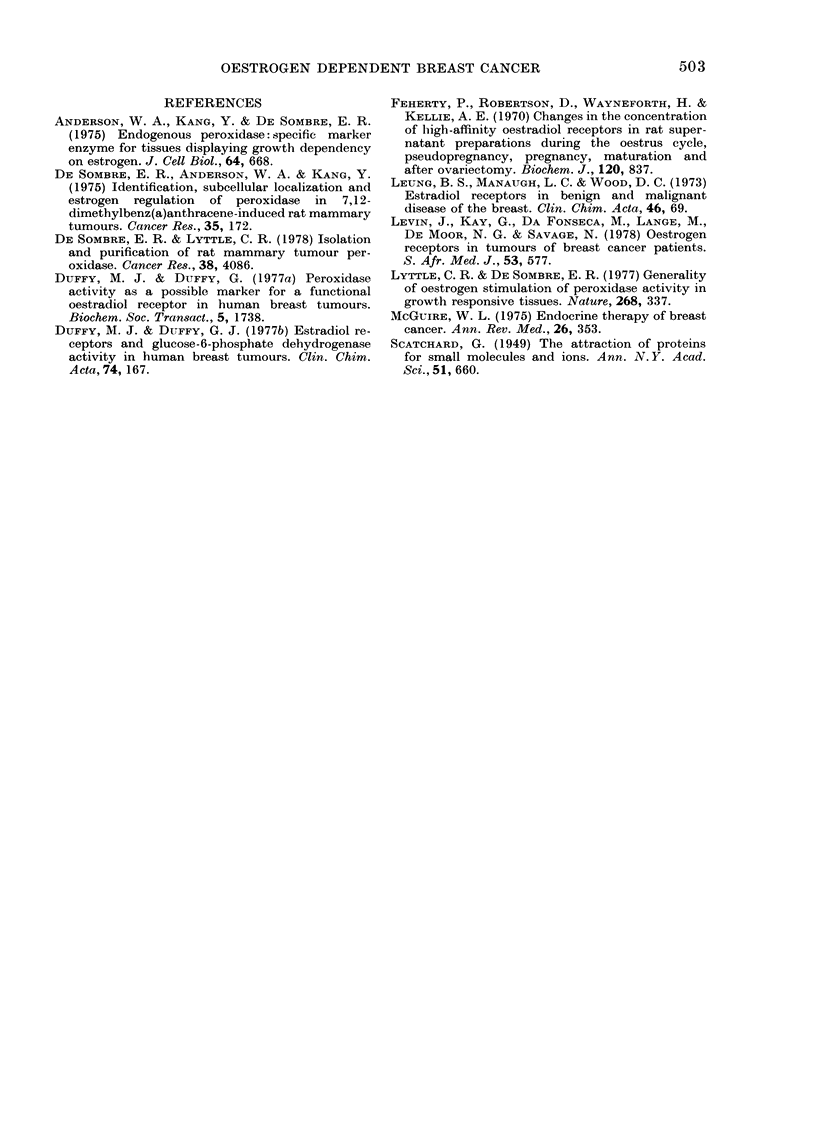# Peroxidase as a marker for oestrogen dependence in human breast cancer.

**DOI:** 10.1038/bjc.1979.208

**Published:** 1979-09

**Authors:** J. R. Collings, N. Savage


*Br. J. Cancer* (1979) **40**, 500

***Short Communication***

### PEROXIDASE AS A MARKER FOR OESTROGEN DEPENDENCE IN HUMAN BREAST CANCER

J. R. COLLINGS AND N. SAVAGE

*From the Department of Medical Biochemistry, University of Witwatersrand Medical School, Johannesburg 2001, South Africa*

Received 27 February 1979 Accepted 18 May 1979

LEVELS of oestradiol receptors (ER) in human breast cancer are routinely assayed in many centres as a prognostic guide to the postoperative hormonal control of metastases. Between 50% and 70% of primary tumours contain ER, and of these, 50–60% respond favourably to endocrine therapy (McGuire, 1975). About half of the $ER^+$ tumours fail to respond to hormonal manipulations. The reason for this is unclear. It is conceivable that the receptors are defective in a way which renders them incapable of mediating the effects of the hormone. Such a defect might exist at the level of transformation, translocation or chromatin binding of the oestradiol–ER complex. Consequently, detection of a gene product of oestrogen action in conjunction with the measurement of ER could provide a more reliable index of the hormone responsiveness of a tumour than the presence of ER alone.

The enzyme peroxidase (E.C. 1.11.1.7) is a possible candidate for this role, since it has been demonstrated in rat and human mammary tumours (De Sombre *et al.*, 1975; Lyttle & De Sombre, 1977) and oestrogen administration has been shown to induce the enzyme in oestrogen-sensitive tissues (Lyttle & De Sombre, 1977) Thus, peroxidase has been suggested as a marker for tissues showing growth dependence on oestrogens (Anderson *et al.*, 1975). In this study the presence of peroxidase was correlated with the presence of ER in 130 breast cancers.

Breast cancer tissue removed at operation was immediately frozen and stored in liquid $N_2$ until assayed. Since tumours might not be uniform throughout their mass, the same sample of tissue was used for both the ER and peroxidase determinations. All procedures were carried out at 4°C unless otherwise stated.

Thawed tissue was chopped finely and homogenized in 4 vol of ice-cold TED buffer (1·5mM ethylenediaminetetra-acetic acid; 0·5mM dithiothreitol; 0·01M Tris-HCl buffer at pH 7·4) by means of 3 × 20-sec bursts with an Ultra-Turrax homogenizer. The homogenate was centrifuged for 1 h at 50,000 *g*. The supernatant was assayed for ER, while the pellet was extracted for measurement of peroxidase activity.

ER content was determined by the method of Levin *et al.* (1978). 200 μl aliquots of supernatant were incubated in the presence of 5 different concentrations of [$^3$H] oestradiol (20–100 pg/ml) in a final volume of 500 μl for 18 h at 4°C.

500 μl of cold TED buffer containing 0·0025% dextran and 0·25% charcoal (DCC) were then added to each tube; the tubes were vortexed and incubated at 4°C for 90 min. The DCC was pelleted at 1,000 g for 15 min and 500 μl of supernatant counted in a Packard Tricarb Spectrometer. A 5-point Scatchard plot (Scatchard, 1949) was constructed for each sample and the binding affinity for oestradiol ($K_d$) and the total number of receptors (expressed as fmol [$^3$H]-oestradiol bound/mg cytosol protein) calculated. Competitive binding analysis (Leung *et al.*, 1973) was also carried out for each sample to establish the oestradiol-binding index. The receptor assay was regarded as positive if the results showed a Scatchard plot, a $K_d < 10^{-10}$M, > 3fmol [$^3$H] oestradiol bound/mg protein and a binding index > 12%.

*Peroxidase assay.*—The pellet from the initial 50,000 $g \times 60$ min spin was resuspended in 4 vol of ice-cold hypertonic extraction buffer (1·2M NaCl; 0·01M Tris-HCl buffer, pH 7·2) by means of 3 × 20-sec bursts with an Ultra-Turrax homogenizer. The suspension was centrifuged at 39,000 $g$ × 45 min to obtain the supernatant containing solubilized peroxidase. Dithiothreitol is known to inhibit peroxidase in a reversible fashion (De Sombre & Lyttle, 1978). Control experiments in which dithiothreitol was omitted from the TED buffer showed a negligible residual effect of the dithiothreitol on the solubilized peroxidase. The peroxidase activity of the hypertonic supernatant was assayed by the method of Lyttle & De Sombre (1977). Between 20 and 200 $\mu$l of hypertonic supernatant was added to the reaction mixture (0·013M guaiacol, 1·2M NaCl, 0·33mM $H_2O_2$, 0·01M Tris-HCl at pH 7·2) in a final volume of 3 ml. The reaction rate at 30°C was followed by recording the absorbance change at 470 nm in a Gilford Spectrophotometer against a blank from which $H_2O_2$ was omitted. Peroxidase activity was expressed as nmol tetraguaiacol formed/mg protein/min. Protein concentrations were determined by the Lowry method, with bovine serum albumin as standard. Using the above method, more than 95% of the total peroxidase activity was found in the hypertonic supernatant, and only minimal amounts (< 5%) associated with the original cytosol and residual particulate fractions. Normal and hyperplastic breast tissue (obtained from women undergoing cosmetic surgery) possessed negligible peroxidase activity. Thus the peroxidase assay was considered positive at the lowest level distinguishable from the blank (1·0 nmol tetraguaiacol formed/mg protein/min).

TABLE I—*Distribution of peroxidase activity in breast tumours with and without ER*

| Receptor status | No. of tumours | % Peroxidase$^+$ |
|---|---|---|
| ER$^+$ | 78 | 65 |
| ER$^-$ | 52 | 71 |

Using the criteria described above, ER was found in 60% of tumours (Table I). 65% of ER$^+$ tumours showed detectable peroxidase activity while 71% of the ER$^-$ tumours were peroxidase$^+$. Thus no correlation was found between the presence of ER and peroxidase activity. The data were further analysed in terms of the histological diagnosis of tumour type. Three main categories of tumours were considered: adenocarcinoma, duct-cell carcinoma and undifferentiated or anaplastic carcinoma. There were no significant differences in ER and peroxidase distributions among the 3 groups (Table II). In addition, some tumours showing heavy mononuclear-cell infiltration (medullary carcinomas) were peroxidase$^-$. It is therefore unlikely that the finding of peroxidase activity in the absence of ER is explicable solely by infiltration by lymphocytes or other cell types.

TABLE II.—*Histology, receptor status and peroxidase activity in* 43 *breast tumours*

| Tumour type | No. of tumours | % Peroxidase$^+$ tumours |
|---|---|---|
| ER$^+$ adenocarcinoma | 14 | 71 |
| ER$^-$ ,, | 5 | 60 |
| ER$^+$ duct-cell carcinoma | 7 | 57 |
| ER$^-$ ,, ,, ,, | 5 | 60 |
| ER$^+$ anaplastic carcinoma | 10 | 50 |
| ER$^-$ ,, ,, | 2 | 100 |

Recently, a fairly good correlation between the presence of ER and peroxidase activity in human treast-carcinoma tissue has been reported (Duffy & Duffy, 1977*a*). These workers found that 78% of

FIG. 1.—Distribution of peroxidase activity in 85 peroxidase$^+$ tumours. Shaded areas represent the percentage of ER$^+$ tumours in each group.

ER$^+$ tumours showed peroxidase activity, whilst only 20% of ER$^-$ tumours were peroxidase$^+$. Our results do not support these findings. In our study, 65% of ER$^+$ tumours were peroxidase$^+$ whilst 71% of ER$^-$ tumours had peroxidase activity (Table I). This discrepancy is unlikely to be due to different criteria for peroxidase positivity, as 63% of tumours with high levels of peroxidase activity were ER$^-$ as opposed to 30% of tumours at lower levels of peroxidase activity (see Fig. 1). However, it could be due to differences in the procedures used to assay receptors (Duffy & Duffy, 1977*b*; Feherty *et al.*, 1970). These workers incubated cytosol with [$^3$H]oestradiol at 30°C for 30 min, whereas in our study cytosol was incubated with [$^3$H]oestradiol at 4°C for 18 h. Also, we have adopted rather stringent criteria for establishing a positive ER assay. Thus, an ER assay was considered positive if the results showed a Scatchard plot, a Kd of $< 10^{-10}$M, a binding index of $> 12\%$ and if $> 3$fmol [$^3$H]oestradiol was bound/mg cytosol protein. Duffy and Duffy (1977*b*) considered an assay positive if $> 10$fmol [$^3$H]oestradiol were bound/mg protein, but had no other criteria.

There are several possible explanations for the finding of high levels of peroxidase activity in ER$^-$ tumours. Our method of receptor assay measures only cytoplasmic receptors; it is conceivable that when relatively high levels of endogenous oestrogens are present in the tissues, nuclear translocation of the hormone–receptor complexes caused depletion of the ER pool and a negative ER assay. Alternatively, peroxidase might be induced by other hormones or by a mechanism independent of hormone action. Thirdly, it is possible that there are several types of peroxidase in the tissue, only one of which is induced by oestradiol. These possibilities are at present under investigation.

The true value of the peroxidase assay in assessing the hormone responsiveness of a breast cancer will only emerge when it is possible to correlate enzyme activity with response to endocrine therapy. Clinical follow-up studies of patients on such therapy are under way and we expect to report on this in the future.

J.R.C. is grateful to the Medical Research Council of South Africa for the award of a post-graduate bursary.

We are indebted to Professor N. G. de Moor, Department of Radiotherapy and Mr M. Lange, Department of Surgery, University of the Witwatersrand, for the supply of tumour tissue.

### REFERENCES

ANDERSON, W. A., KANG, Y. & DE SOMBRE, E. R. (1975) Endogenous peroxidase: specific marker enzyme for tissues displaying growth dependency on estrogen. *J. Cell Biol.*, **64,** 668.

DE SOMBRE, E. R., ANDERSON, W. A. & KANG, Y. (1975) Identification, subcellular localization and estrogen regulation of peroxidase in 7,12-dimethylbenz(a)anthracene-induced rat mammary tumours. *Cancer Res.*, **35,** 172.

DE SOMBRE, E. R. & LYTTLE, C. R. (1978) Isolation and purification of rat mammary tumour peroxidase. *Cancer Res.*, **38,** 4086.

DUFFY, M. J. & DUFFY, G. (1977*a*) Peroxidase activity as a possible marker for a functional oestradiol receptor in human breast tumours. *Biochem. Soc. Transact.*, **5,** 1738.

DUFFY, M. J. & DUFFY, G. J. (1977*b*) Estradiol receptors and glucose-6-phosphate dehydrogenase activity in human breast tumours. *Clin. Chim. Acta*, **74,** 167.

FEHERTY, P., ROBERTSON, D., WAYNEFORTH, H. & KELLIE, A. E. (1970) Changes in the concentration of high-affinity oestradiol receptors in rat supernatant preparations during the oestrus cycle, pseudopregnancy, pregnancy, maturation and after ovariectomy. *Biochem. J.*, **120,** 837.

LEUNG, B. S., MANAUGH, L. C. & WOOD, D. C. (1973) Estradiol receptors in benign and malignant disease of the breast. *Clin. Chim. Acta*, **46,** 69.

LEVIN, J., KAY, G., DA FONSECA, M., LANGE, M., DE MOOR, N. G. & SAVAGE, N. (1978) Oestrogen receptors in tumours of breast cancer patients. *S. Afr. Med. J.*, **53,** 577.

LYTTLE, C. R. & DE SOMBRE, E. R. (1977) Generality of oestrogen stimulation of peroxidase activity in growth responsive tissues. *Nature*, **268,** 337.

MCGUIRE, W. L. (1975) Endocrine therapy of breast cancer. *Ann. Rev. Med.*, **26,** 353.

SCATCHARD, G. (1949) The attraction of proteins for small molecules and ions. *Ann. N.Y. Acad. Sci.*, **51,** 660.